# Supplementary material for: A pathway to coexistence of electroluminescence and photovoltaic conversion in organic devices
Source: Nat Commun. 2025 Dec 7;17:694. doi: 10.1038/s41467-025-67332-0 (PMC12819378; doi:10.1038/s41467-025-67332-0)
Supplement: Supplementary file 1 — Supplementary Information [file 41467_2025_67332_MOESM1_ESM.pdf]

SUPPLEMENTARY INFORMATION

A pathway to coexistence of electroluminescence and photovoltaic conversion in organic devices

Taku Oono[1], Yusuke Aoki[2], Tsubasa Sasaki[1], Haruto Shoji[2], Takuya Okada[1], Takahisa Shimizu[1,2], Takuji Hatakeyama[3], Hirohiko Fukagawa[4, *]

[1] Japan Broadcasting Corporation (NHK), Science & Technology Research Laboratories, 1-10-11 Kinuta, Setagaya-ku, Tokyo 157-8510, Japan
[2] Department of Applied Physics, Tokyo University of Science, Tokyo 125-8585, Japan
[3] Department of Chemistry, School of Science, Kyoto University, Sakyo-ku, Kyoto 606-8502, Japan
[4] Center for Frontier Science, Chiba University, 1-33, Yayoicho, Inage-ku, Chiba-shi, Chiba, 263-8522, Japan

[*]Email: hiro.fukagawa@chiba-u.jp, TEL: +81-43-290-3248

Contents of Supplementary Materials

Supplementary Figures
Supplementary Tables
Supplementary References

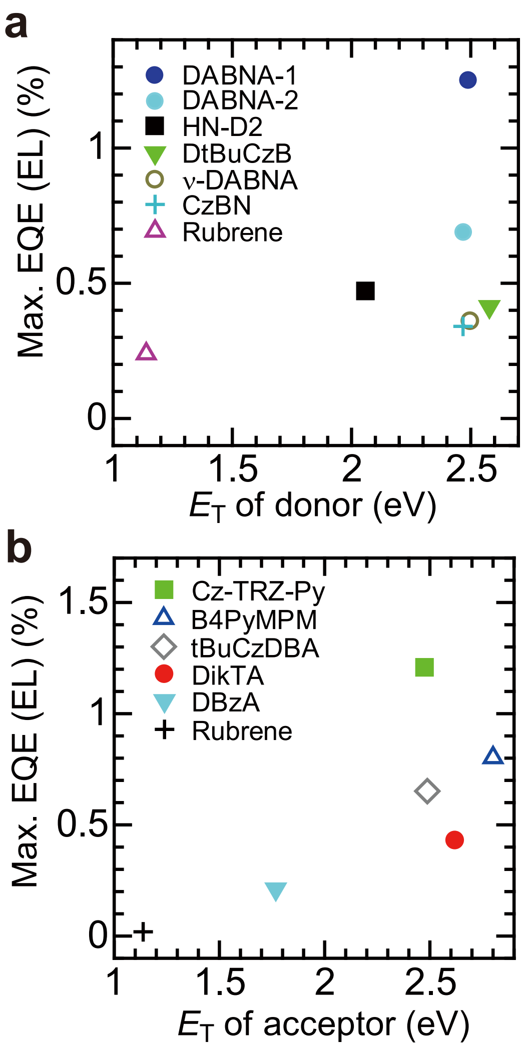

Figure S1. Influence of donor and acceptor triplet energies on EQE_{EL}. Maximum EQE_{EL} of MF devices as a function of (a) E_T of donor (from Fig. 3 in the manuscript) and (b) E_T of acceptor (from Fig. 4 in the manuscript).

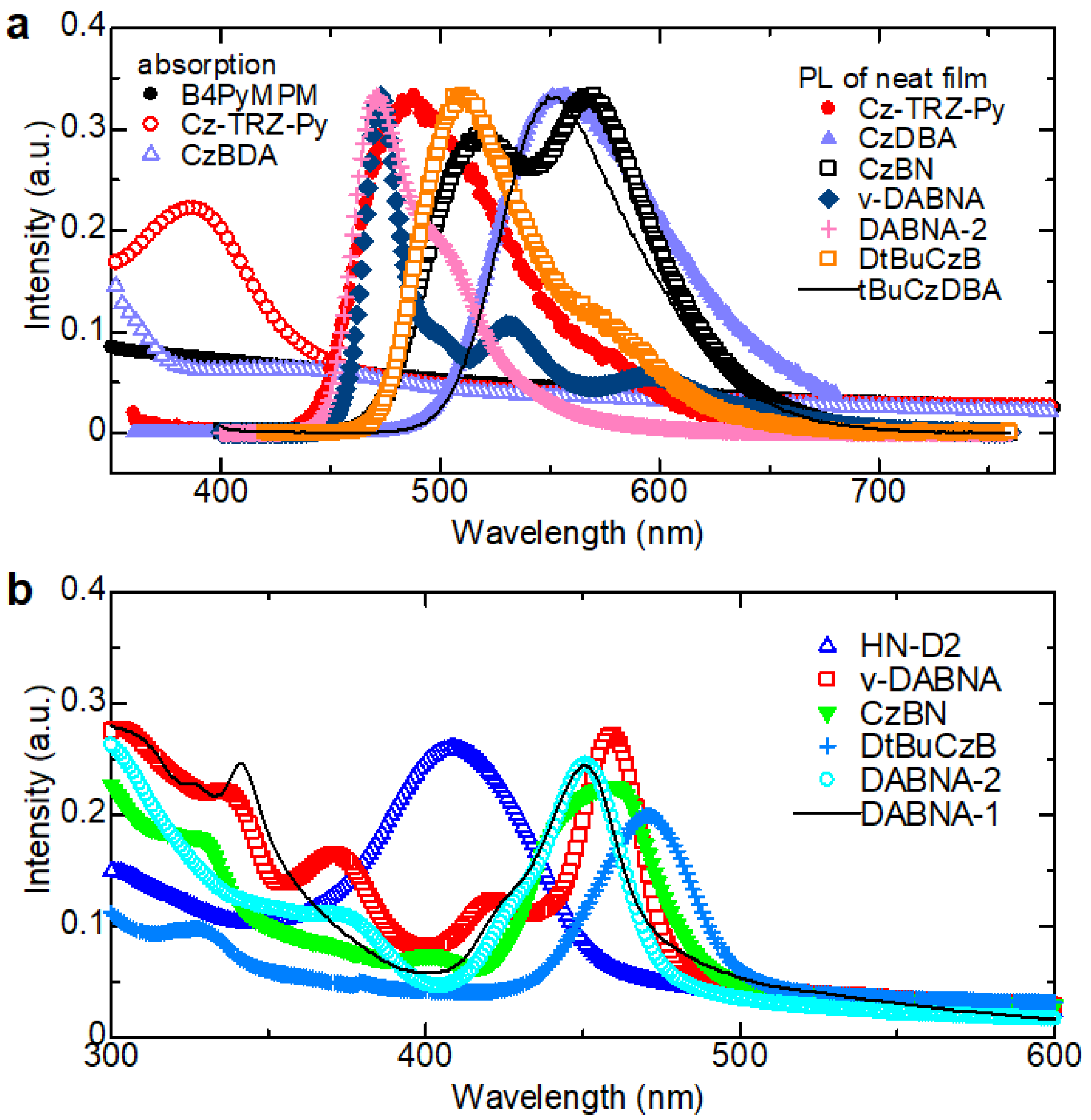

Figure S2. Optical absorption and emission properties of donor and acceptor films. (a) UV−vis absorption and photoluminescence spectra of selected acceptor neat films. (b) UV−vis spectra of donor neat films.

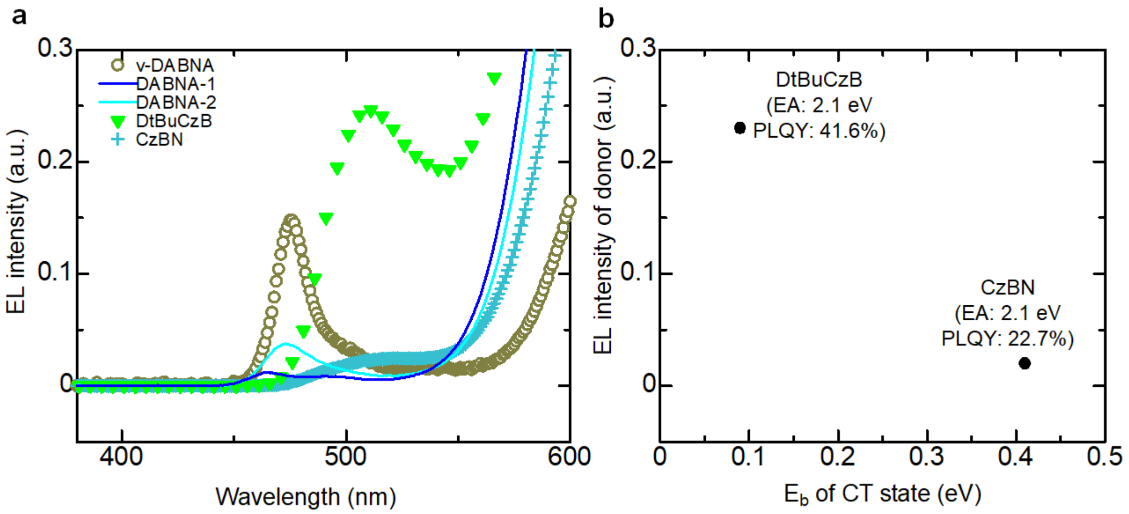

Figure S3. Relationship between EL spectral features and CT-state E_b in MF devices. (a) Magnified view of the EL spectra of MF devices. (b) Correlation between EL intensity of donor and the E_b of CT state in the EL spectrum.

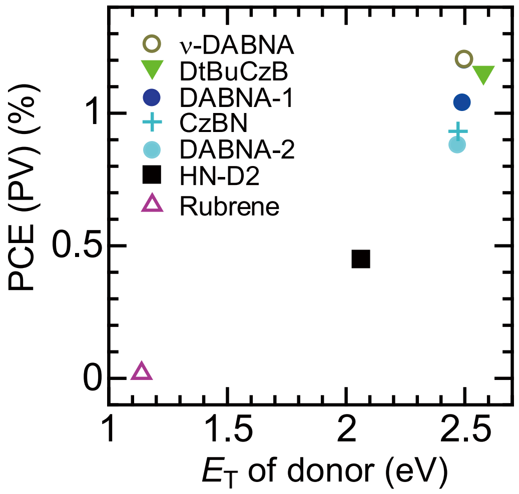

Figure S4. PCE of MF devices as a function of E_T of the donor.

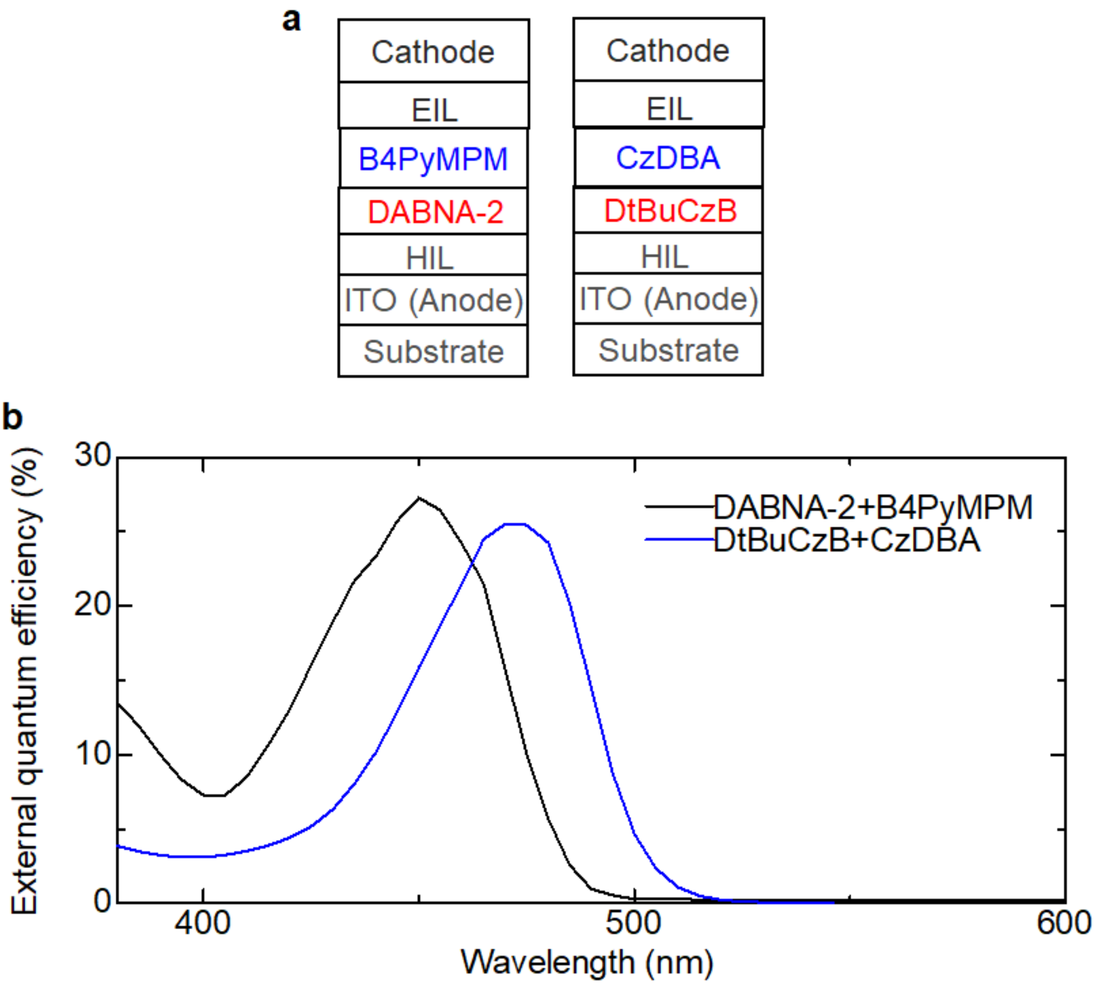

Figure S5. Photovoltaic external quantum efficiency characteristics of MF devices with various donor/acceptor pairs. (a) Schematics of the MF devices used for photovoltaic external quantum efficiency measurements. (b) Photovoltaic external quantum efficiency spectra of MF devices with different donor/acceptor combinations.

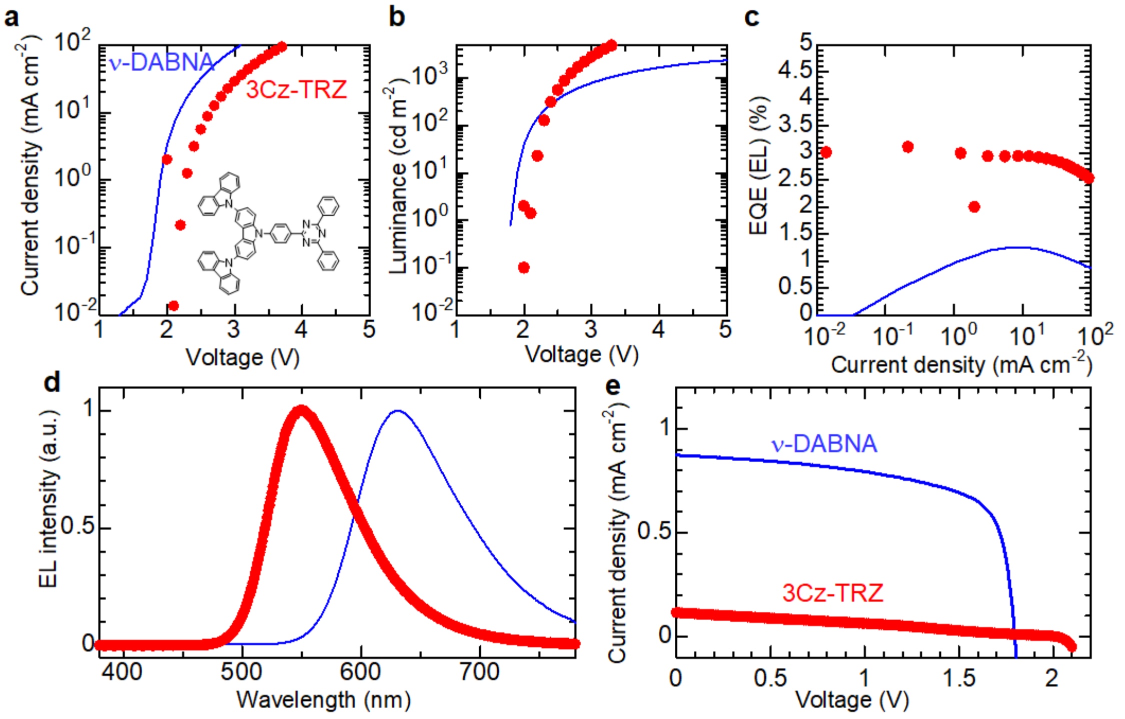

Figure S6. Characteristics of MF devices using 3Cz-TRZ (ITO/HIL/3Cz-TRZ (30 nm)/CzDBA (30 nm)/EIL/Al).[1] (a) Current density–voltage and (b) Luminance–voltage characteristics. (c) EQE traces of devices for electroluminescence. (**d**), Electroluminescence spectra of devices. (**e**) Current–density voltage curves of devices measured under simulated AM1.5G solar illumination.

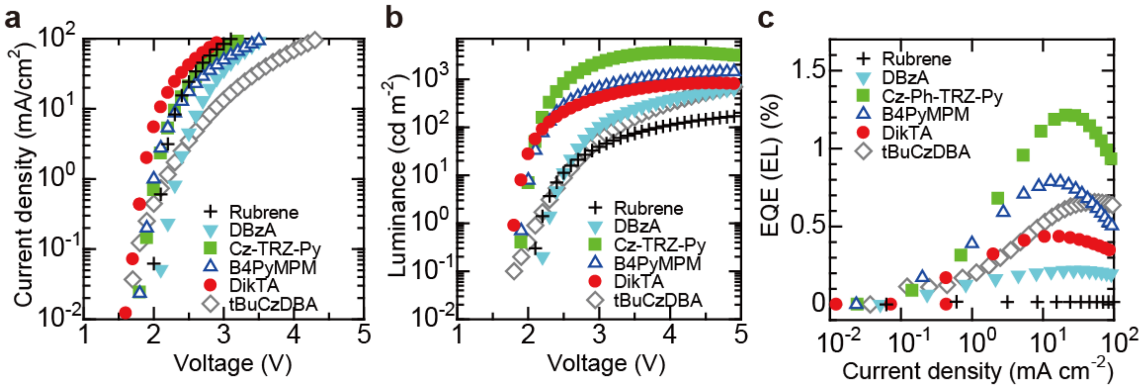

Figure S7. Characteristics of MF devices using ν-DABNA with various acceptors. (a) Current density–voltage and (b) Luminance–voltage characteristics. (c) EQE traces of devices for electroluminescence.

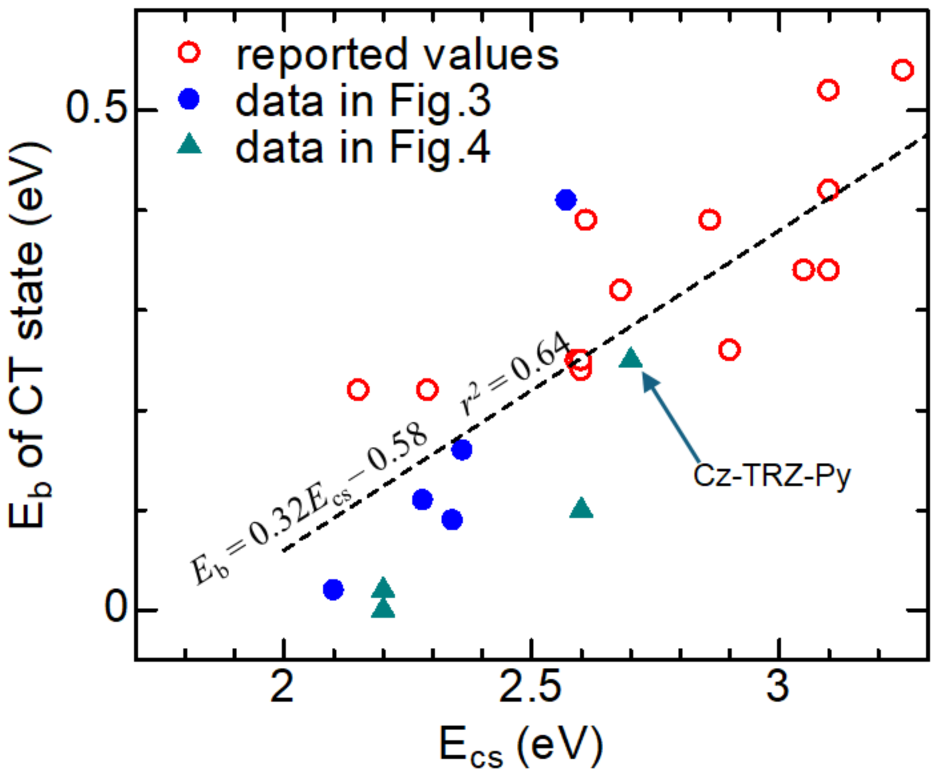

Figure S8. E_b of CT state as a function of E_{CS}. The coefficients of determination (r^2) derived from the linear approximation method are also shown.

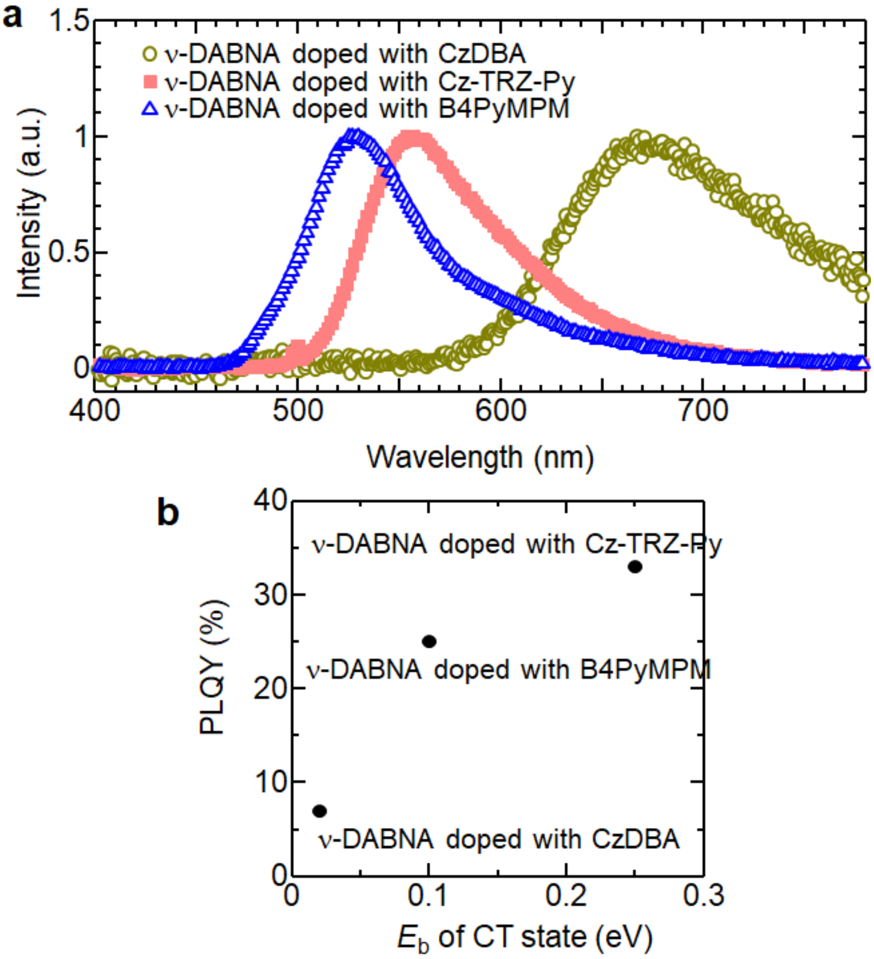

Figure S9. Photoluminescence properties of ν-DABNA-based doped films. (a) Photoluminescence spectra of ν-DABNA doped films (ν-DABNA: acceptor = 50:50). (b) Photoluminescent quantum yield (PLQY) of ν-DABNA doped films as a function of E_b of CT state.

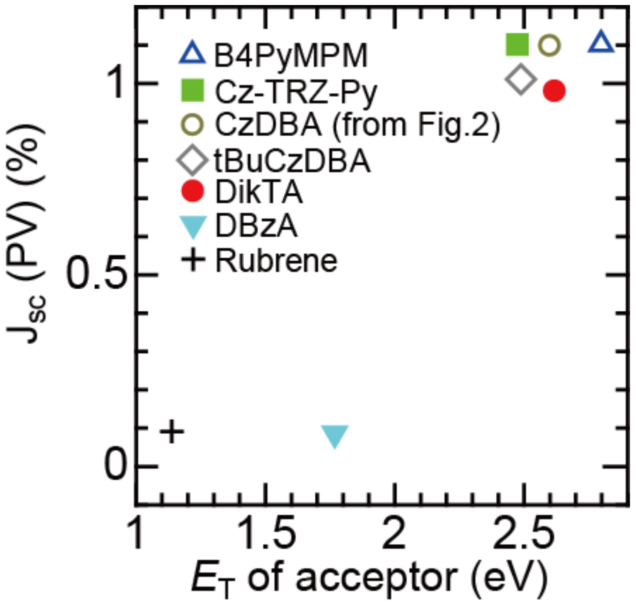

Figure S10. ***J*SC of MF devices as a function of *E*T of the acceptor.**

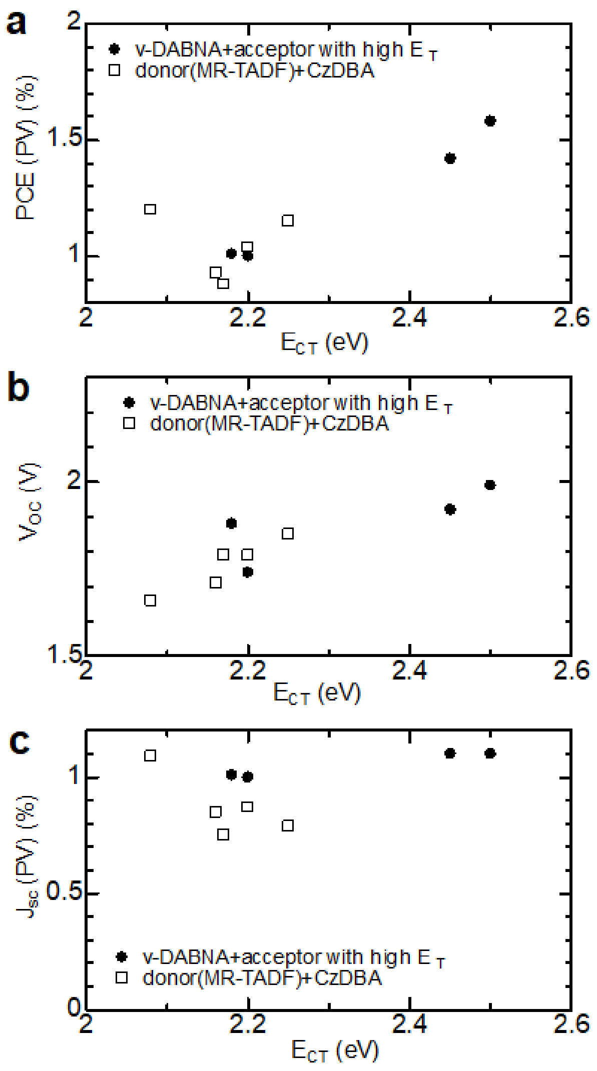

Figure S11. Correlations between photovoltaic performance and E_{CT} in MF devices. (a) PCE_{PV} as a function of E_{CT} of MF devices using MR-TADF material. (b) V_{OC} as a function of E_{CT} of MF devices. (c) J_{SC} as a function of E_{CT} of MF devices.

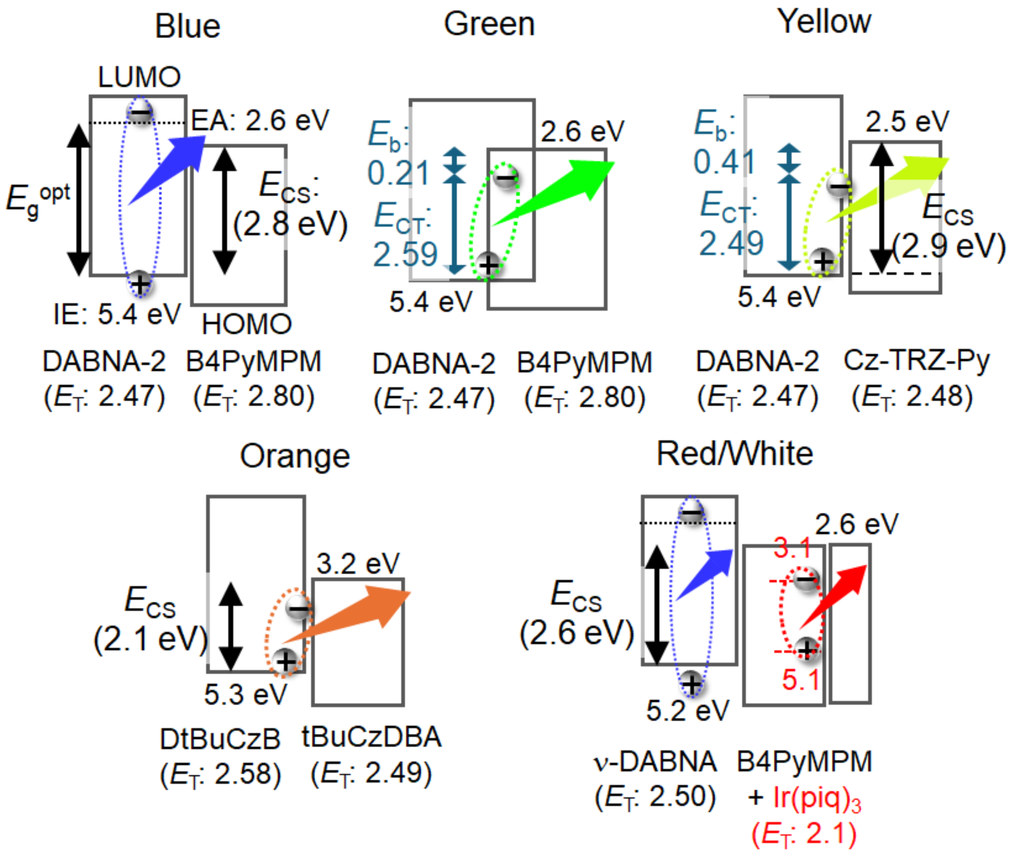

Figure S12. Schematic of energy-level diagram of optimised devices.

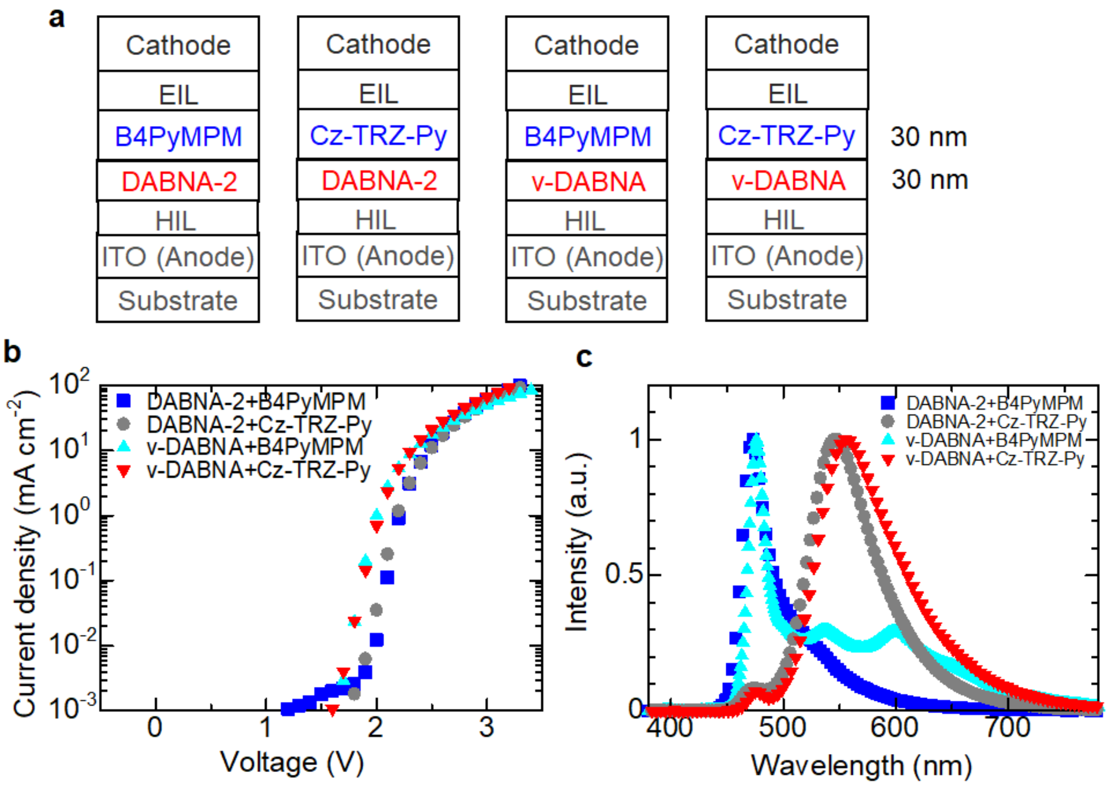

Figure S13. Device structure and emission characteristics of MF devices with different donor/acceptor pairs. (a) Schematic illustration of MF devices with various donor/acceptor pairs. (b) Current density–voltage characteristics. (c) Electroluminescence spectra of MF devices operated at current density of 10 mA cm^{-2}.

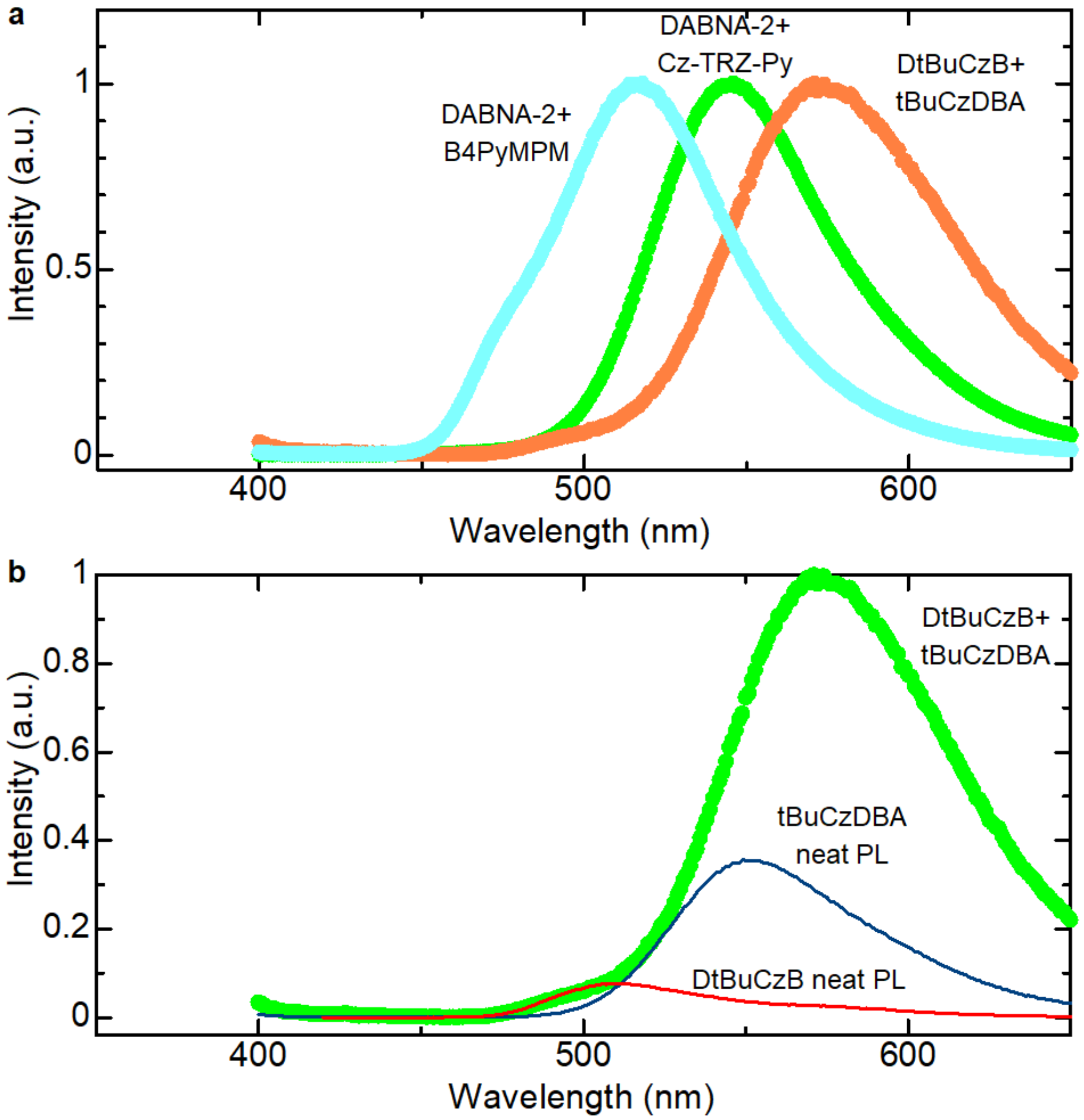

Figure S14. Photoluminescence characteristics of donor–acceptor doped films. (a) Photoluminescence spectra of doped films (donor:acceptor = 50:50). (b) Comparison of the photoluminescence spectrum of a doped film (DtBuCzB and tBuCzDBA) with those of the neat films of DtBuCzB and tBuCzDBA. In the mixed film of DtBuCzB and tBuCzDBA, in addition to exciplex emission, emissions originating from both MR-TADF materials were also observed.

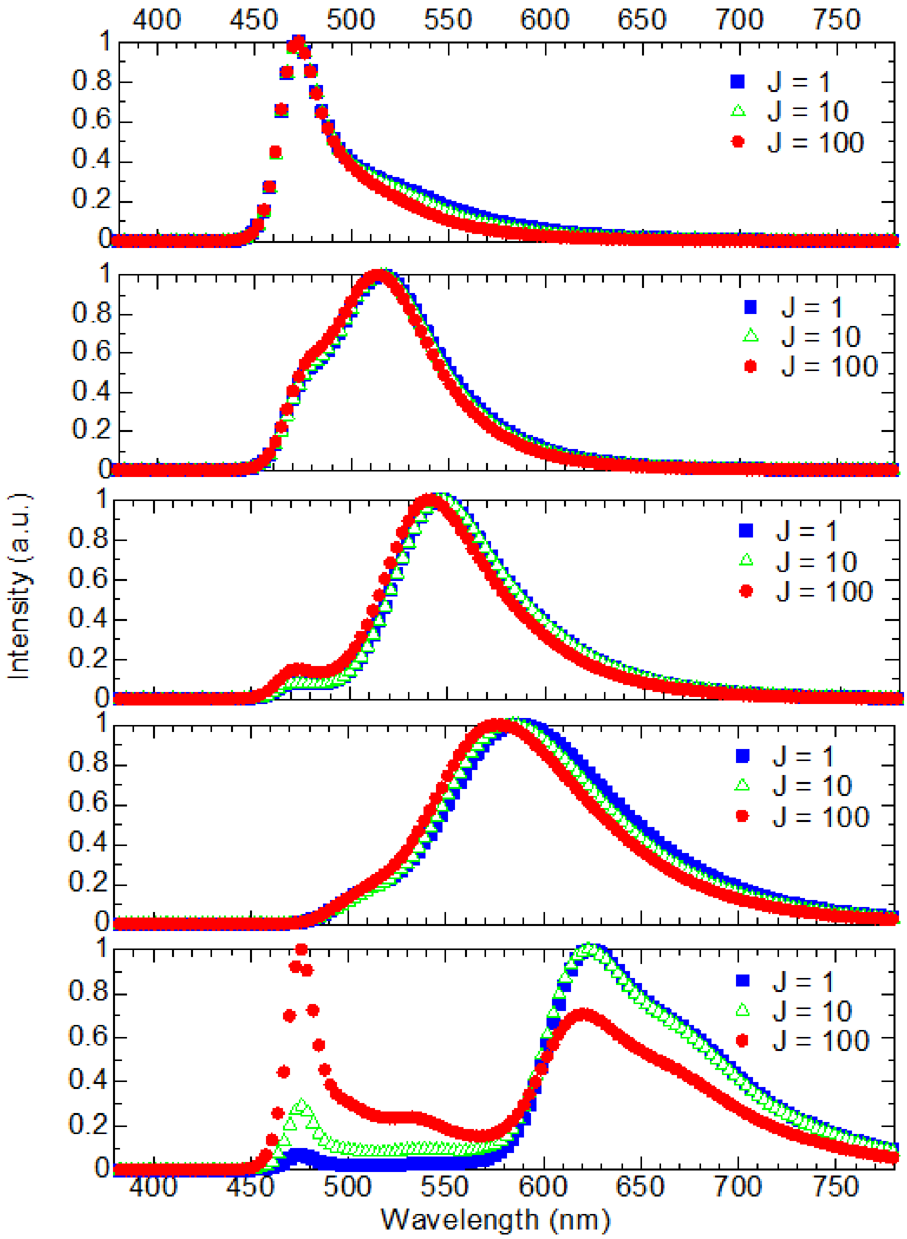

Figure S15. Electroluminescence spectra of MF devices operated at various current densities.

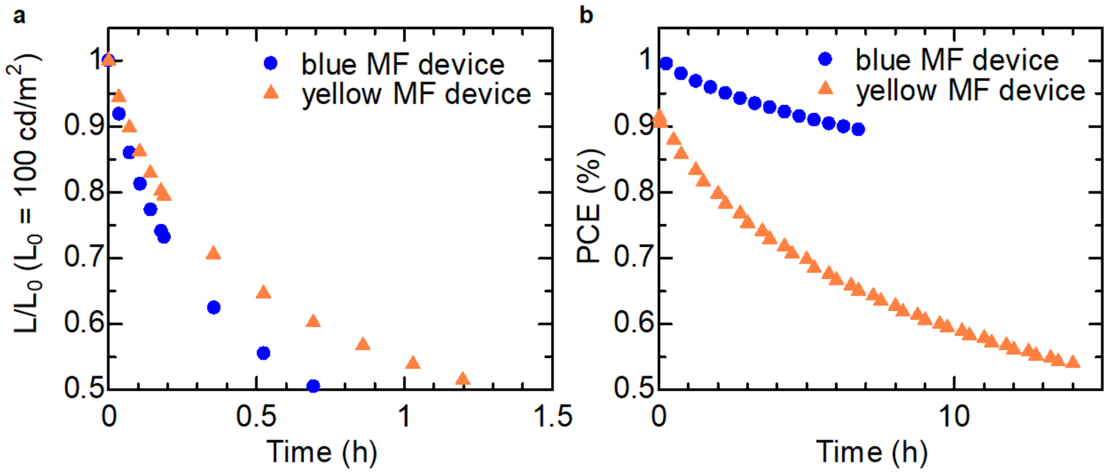

Figure S16. Operational stability of blue and yellow multifunctional devices. (a) Luminance–time characteristics of blue and yellow emitting MF devices under constant dc with initial luminance of 100 cd m^{-2}. (b) Change in PCE of MF devices under continuous illumination (simulated AM1.5G solar, room temperature).

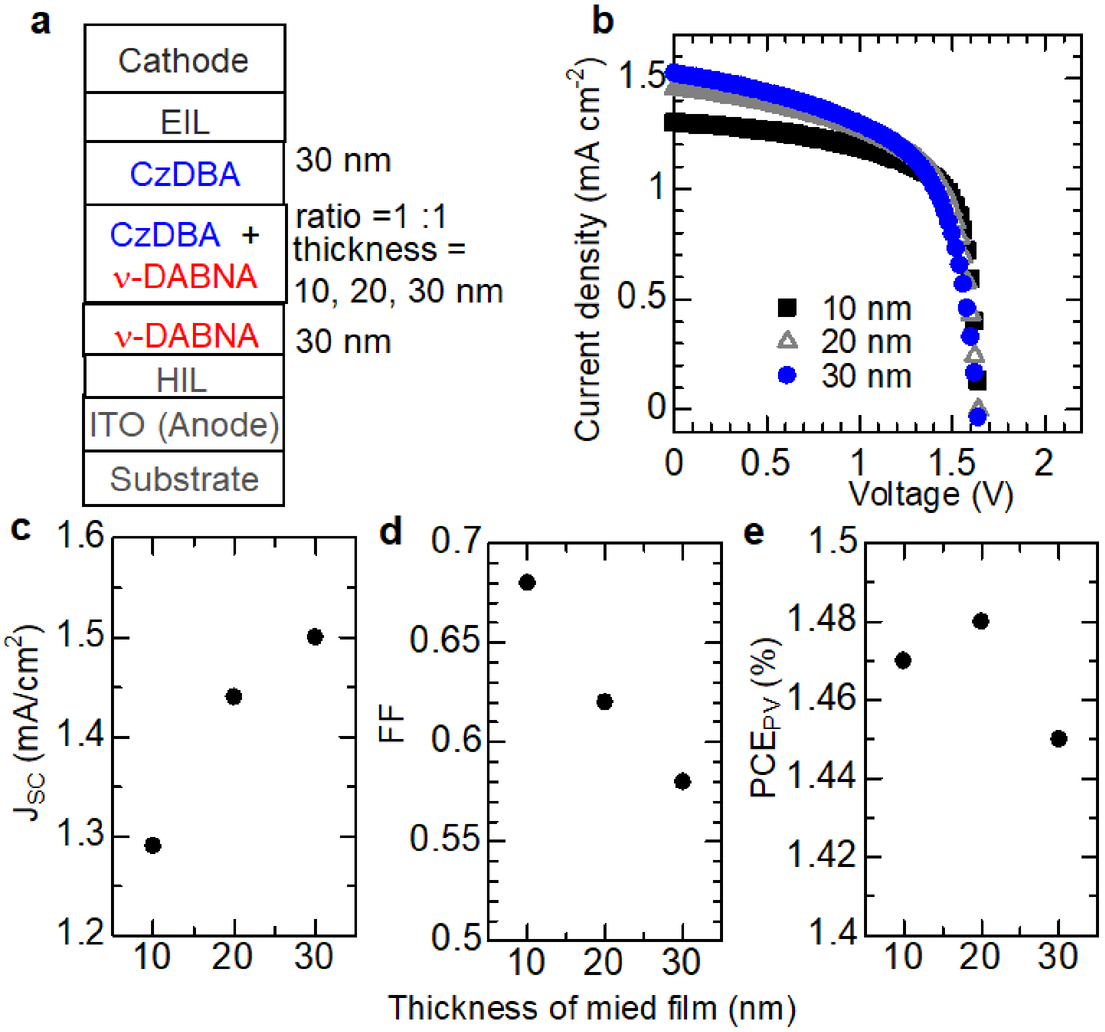

Figure S17. Effect of mixed-film thickness on MF device performance. (a) Schematic illustration of MF devices with three mixed films (thickness: 10, 20, 30 nm). (b) Current density–voltage curves of devices measured under simulated AM1.5G solar illumination. (c) J_{SC} of MF devices as a function of thickness of mixed film. (d) FF of MF devices as a function of thickness of mixed film. (e) PCE_{PV} of MF devices as a function of thickness of mixed film.

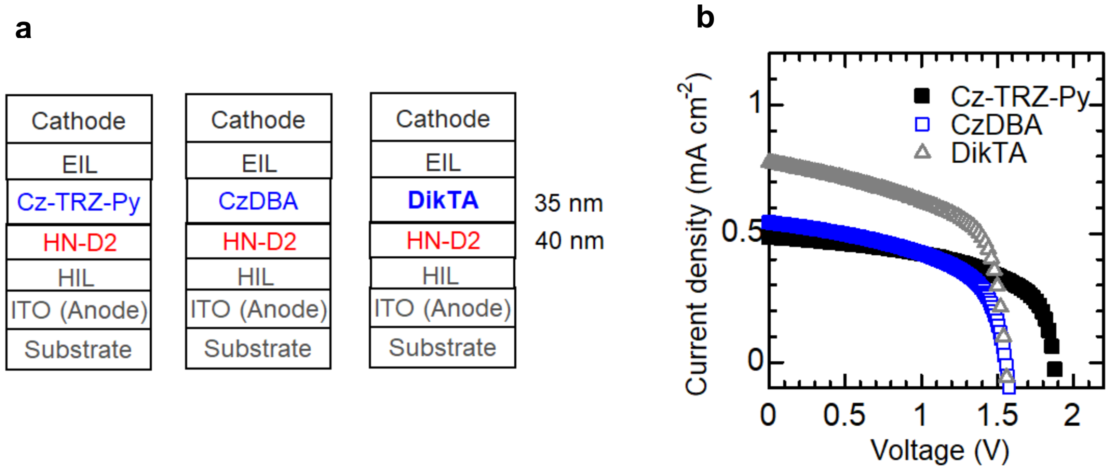

Figure S18. Effect of acceptor selection on photovoltaic performance of MF devices. (a) Schematic illustration of MF devices with various acceptors. (b) Current density–voltage curves of devices measured under simulated AM1.5G solar illumination.

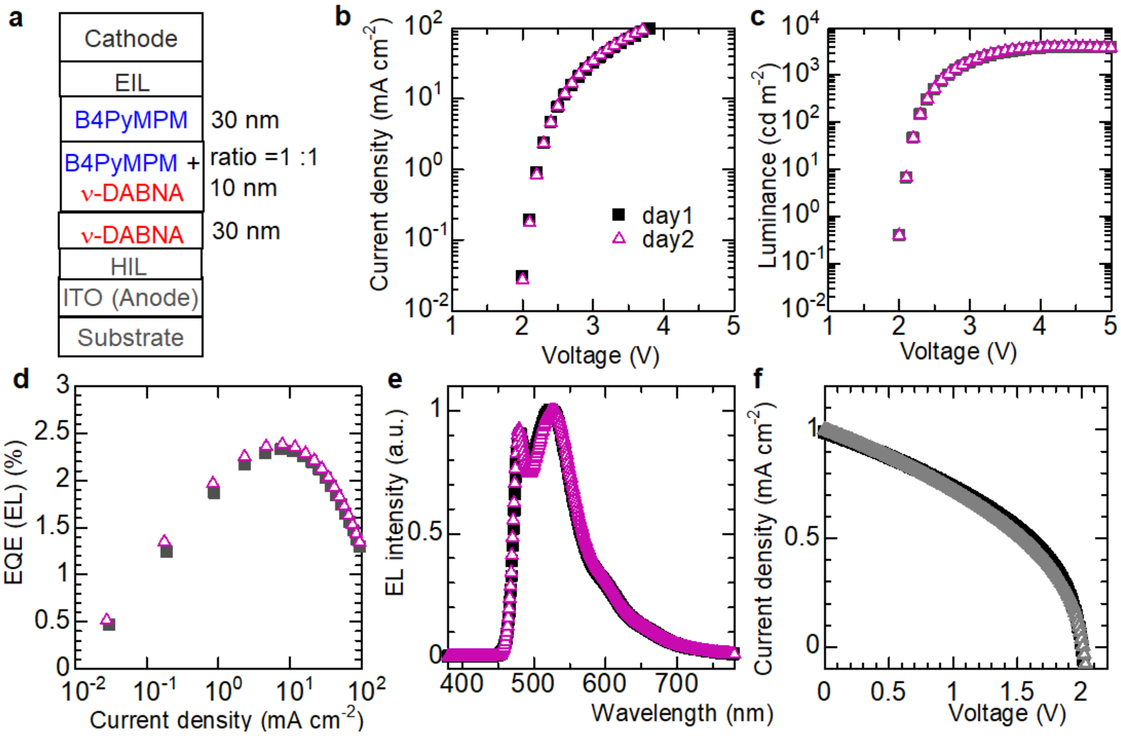

Figure S19. Characteristics of MF devices fabricated on different experimental days. (a) Schematic illustration of the MF device. (b,c) Current density–voltage (b) and luminance–voltage (c) characteristics of devices with different donors. (d), EQE_{EL} traces of devices for electroluminescence. (e), Electroluminescence spectra of devices. (f) Current–density voltage curves of devices measured under simulated AM1.5G solar illumination.

Supplementary Table 1. Summary of energies of materials used for MF devices.

	*IE (eV)	**EA (eV)	***E_T (eV)
DtBuCzB	5.34	2.1	2.58
ν-DABNA	5.2	1.4	2.50
DABNA-1	5.46	1.4	2.49
DABNA-2	5.38	1.4	2.47
CzBN	5.67	2.1	2.47
HN-D2	5.13	1.7	2.06
tBuCzDBA	5.9	3.0	2.49
Cz-TRZ-Py	5.69	2.5	2.48
CzDBA	6.0	3.1	2.60
B4PyMPM	7.0	2.6	2.80
DiKTa	5.93	3.0	2.62
Rubrene	5.4	2.6	1.14

*IE: ionisation energy measured by using AC-3

**EA: electron affinity measured by using LEIPS

***E_T: triplet energy estimated from phosphorescent spectrum

Supplementary Table 2. Summary of energies and characteristics of MF devices.

	donor	acceptor	E_{CT} (eV)	E_{CS} (eV)	E_b (eV)	J_{SC} (mA/cm^2)	V_{OC} (V)	FF	PCE_{PV} (%)	Max. EQE_{EL} (%)
Fig. 3	DtBuCzB	CzDBA	2.25	2.34	0.09	0.79	1.85	0.78	1.15	0.41
Fig. 3	v-DABNA	CzDBA	2.08	2.1	0.02	1.09	1.66	0.68	1.2	0.36
Fig. 3	DABNA-1	CzDBA	2.2	2.36	0.16	0.87	1.79	0.67	1.04	1.25
Fig. 3	DABNA-2	CzDBA	2.17	2.28	0.11	0.75	1.79	0.66	0.88	0.69
Fig. 3	CzBN	CzDBA	2.16	2.57	0.41	0.85	1.71	0.64	0.93	0.34
Fig. 3	HN-D2	CzDBA	1.96	2.03	0.07	0.53	1.55	0.54	0.45	0.47
Fig. 3	Rubrene	CzDBA	-	2.3	-	0.09	1.53	0.18	0.02	0.24
Fig. 4	v-DABNA	tBuCzDBA	2.18	2.2	0.02	1.01	1.88	0.63	1.01	0.65
Fig. 4	v-DABNA	Cz-TRZ-Py	2.45	2.7	0.25	1.10	1.92	0.67	1.42	1.21
Fig. 4	v-DABNA	B4PyMPM	2.5	2.6	0.1	1.10	1.99	0.71	1.58	0.8
Fig. 4	v-DABNA	DiKTa	2.2	2.2	0	1.00	1.74	0.59	1.00	0.43
Fig. 4	v-DABNA	Rubrene	-	2.6	-	0.09	1.85	0.31	0.02	0.02
Fig. 4	v-DABNA	DBzA	-	2.7	-	0.1	2.10	0.2	0.06	0.21

E_{CT}: energy of charge transfer (CT) state

E_{CS}: final charge-separated (CS) state energy

E_b: exciton binding energy of CT state

PCE_{PV}: power conversion efficiency of photovoltaic

Max. EQE_{EL}: external quantum efficiency (EQE) of EL emission

Supplementary Table 3. Summary of photoluminescent quantum yield (PLQY) of neat and doped films.

Film (ratio, %)	PLQY (%)
DABNA-2 (100, neat film)	44
DABNA-2: Cz-TRZ-Py (50:50)	57
DABNA-2: B4PyMPM (50:50)	44
ν-DABNA (100, neat film)	12
ν-DABNA: Cz-TRZ-Py (50:50)	33
ν-DABNA: B4PyMPM (50:50)	26
ν-DABNA: CzDBA (50:50)	7

Supplementary Table 4. Summary of EL characteristics of MF devices with various emitting colours.

	L_{max} (cd/m^2)	PE_{max} (lm/W)	CE_{max} (cd/A)	Max. EQE_{EL} (%)	CIE (x,y) CCT (J = 1)	CIE (x,y) CCT (J = 10)	CIE (x,y) CCT (J = 100)
Blue	1,477	5.4	4.0	2.11	0.18, 0.31	0.16, 0.29	0.15, 0.26
Green	8,557	35.3	25.9	8.63	0.22, 0.55	0.22, 0.55	0.20, 0.54
Yellow	3,754	25.0	17.6	4.94	0.27, 0.54	0.26, 0.54	0.26, 0.54
Orange	9,877	32.0	22.0	8.69	0.38, 0.57	0.37, 0.57	0.35, 0.57
Red/White	2,050	2.26	1.6	1.59	0.64, 0.33 –	0.57, 0.34 1,827 K	0.42, 0.34 2799 K

L_{max}: maximum luminance

PE_{max}: maximum power efficiency

CE_{max}: maximum current efficiency

CCT: correlated colour temperature

Max. EQE_{EL}: external quantum efficiency (EQE) of EL emission

Supplementary Table 5. Summary of PV characteristics of MF devices with various emitting colours.

	J_{SC} (mA/cm^2)	V_{OC} (V)	FF	PCE_{PV} (%)
Blue	0.83	2.10	0.59	1.03
Green	0.53	2.10	0.41	0.45
Yellow	0.86	2.04	0.52	0.92
Orange	0.73	1.93	0.40	0.56
Red/White	0.97	1.97	0.67	1.28

PCE_{PV}: power conversion efficiency of photovoltaic

Supplementary Table 6. Summary of characteristics of previously reported MF devices.

Material used for MF device	J_{SC} (mA/cm^2)	V_{OC} (V)	FF	PCE_{PV} (%)	Max. EQE_{EL} (%)	emission colour	Ref
rubrene/C60	5.3	0.94	0.56	2.9	< 0.001	Orange	2
rubrene/C60	~2.0	~0.9	-	~2.0	< 0.001	Orange	3
rubrene/ PTCDI-C13	0.33	0.97	0.68	0.15	< 0.001	Orange	4
P3HT + PCBM /rubrene	3.32	0.62	0.51	1.05	< 0.001	Orange	5
BF-DPB/ B4PyMPM	0.6	2.04	0.7	~0.8	1.5	Orange	6,7
DHNRs (CdS, CdSe and ZnSe)	0.36	1.49	0.37	0.20	8.0	Red	8

PCE_{PV}: power conversion efficiency of photovoltaic

Max. EQE_{EL}: external quantum efficiency (EQE) of EL emission

Supplementary References

1. Hirata, S. *et al.* Highly efficient blue electroluminescence based on thermally activated delayed fluorescence. *Nature Mater* **14**, 330–336 (2015).

2. Pandey, A. K. & Nunzi, J.-M. Rubrene/Fullerene Heterostructures with a Half-Gap Electroluminescence Threshold and Large Photovoltage. *Advanced Materials* **19**, 3613–3617 (2007).

3. Engmann, S. *et al.* Higher order effects in organic LEDs with sub-bandgap turn-on. *Nat Commun* **10**, 227 (2019).

4. Yamada, M., Naka, S. & Okada, H. Light-emitting Organic Photovoltaic Devices Based on Rubrene/PTCDI-C13 Stack. *Electrochemistry* **85**, 280–282 (2017).

5. Lou, Y., Wang, Z., Naka, S. & Okada, H. Bi-functional electroluminescent and photovoltaic devices based on rubrene-doped poly(3-hexylthiophene):1-(3-methoxycarbonyl)-propyl-1-phenyl-(6,6)C61 blends. *Synthetic Metals* **162**, 281–284 (2012).

6. Jia, X. *et al.* Molecularly induced order promotes charge separation through delocalized charge-transfer states at donor–acceptor heterojunctions. *Materials Horizons* **11**, 173–183 (2024).

7. Ullbrich, S. *et al.* Emissive and charge-generating donor–acceptor interfaces for organic optoelectronics with low voltage losses. *Nat. Mater.* **18**, 459–464 (2019).

8. Oh, N. *et al.* Double-heterojunction nanorod light-responsive LEDs for display applications.

Science **355**, 616–619 (2017).
